# Central Precocious Puberty and Response to GnRHa Therapy in Children with Cerebral Palsy and Moderate to Severe Motor Impairment: Data from a Longitudinal, Case-Control, Multicentre, Italian Study

**DOI:** 10.1155/2017/4807163

**Published:** 2017-07-16

**Authors:** Patrizia Bruzzi, Maria Francesca Messina, Alessandra Bartoli, Barbara Predieri, Laura Lucaccioni, Simona Filomena Madeo, Alberto Verrotti, Filippo De Luca, Lorenzo Iughetti

**Affiliations:** ^1^Department of Medical and Surgical Sciences of Mothers, Children and Adults, Paediatric Unit, University of Modena & Reggio Emilia, Via del Pozzo, No. 71, 41124 Modena, Italy; ^2^Department of Paediatrics, University of Messina, Padiglione NI Policlinico Universitario, Via Consolare Valeria, 98125 Messina, Italy; ^3^Department of Pediatrics, University of Aquila, Via Vetoio (Coppito 2), 67100 Coppito, Italy

## Abstract

**Background:**

Children affected by neurodevelopmental disability could experience early pubertal changes at least 20 times more than the general population. Limited data about central precocious puberty (CPP) among children affected by cerebral palsy (CP) are available.

**Methods:**

This is a longitudinal, observational, retrospective, case-control study involving 22 children affected by CPP and CP (group A), 22 paired with CP but without CPP (group B), and 22 children with CPP without CP. Auxological, biochemical, and instrumental data were collected at diagnosis of CPP and at 2 follow-up visits.

**Results:**

No differences were detected between groups A (at baseline) and B. At diagnosis of CPP, height SDS adjusted for target height (H-TH SDS) was significantly reduced in A than in C (−0.63 ± 1.94 versus 1.56 ± 1.38), while basal LH and oestradiol levels were significantly elevated in A than in C. During follow-up, despite an effective treatment, growth impairment deteriorated in A than in C (Δ H-SDS from diagnosis of CPP to last follow-up: −0.49 ± 0.91 versus 0.21 ± 0.33, *p* = 0.023).

**Conclusions:**

Diagnosis of CPP could be partially mislead in CP due to growth failure that got worse during follow-up despite therapy. CPP in CP seems to progress rapidly along time supporting the hypothesis of a more intense activation of hypothalamic-pituitary-gonadal-axis in these patients.

## 1. Introduction

Many insults to central nervous system can cause central precocious puberty (CPP), including traumatic brain injury, birth asphyxia, congenital malformation, infections, and several tumours. Moreover, CPP has been also described in a long list of genetic disorders including neurofibromatosis, fragile X syndrome, and tuberous sclerosis. An old-fashioned study published in 1999 reviewed records of 15719 patients with neurodevelopmental disabilities to detect diagnostic data for premature sexual development: CPP was identified in 32 children, with the earliest changes seen in one girl at 1 year and 7 months of age with a primary diagnosis of cerebral palsy (CP) [1]. These first data documented that children with a neurodevelopmental disability are 20 times more at risk of premature pubertal changes when compared to general population. CP, also called nonprogressive childhood encephalopathy, is a common and lifelong condition caused by a brain lesion occurred during structural and functional maturation of the brain in the pre-, peri-, or postnatal period. Despite the high rate of cumulative incidence of CP (estimated 2–2.7 cases for every 1000 live births) [2], the number of studies analysing CPP in CP is limited [3, 4]. In 2002, a survey performed over 207 CP children aged from 3 to 18 years with moderate to severe motor impairment documented that sexual maturation differs in CP from the general population: white girls with CP initiated pubic hair development earlier than the general population, but the age in the onset of breast development was similar and the estimated median age of menarche was even later (about 1.3 years) than for the general population. The same trend was described for white CP boys [4]. Nevertheless, even that survey, still considered the more recent paper published on this topic, did not mention CPP.

The objectives of this study include the detection of specific features of CPP in patients with CP to establish the need of a proper diagnostic and therapeutic approach and to analyse the aetiological complexity of their association.

## 2. Patients and Methods

This is a descriptive, longitudinal, observational, retrospective, case-control study.

The study population was enrolled among children followed up at the paediatric endocrine and neurologic outpatient clinics at three Italian academic centres (Modena, Messina, and Perugia) between January 2001 and December 2014. The sample comprised children with CP and CPP (group A), children with CP without CPP (group B) matched for gender and age with participants of group A, and children with CPP without CP (group C).

None of the children included in this study presented a history of concomitant genetic, metabolic, neurodegenerative disease. Cases of peripheral precocious puberty were ruled out. The mobility and motor impairment of CP patients was classified as levels III, IV, and V according to the Gross Motor Function Classification System (GMFCS) [5].

Anamnestic data comprised gestational and birth information, including birth anthropometric data, comorbidities, and concomitant therapies, especially the use of antiepileptic drugs (AED).

Recorded anthropometric measurements for all participants included length (L) (groups A and B) or standing height (H) (group C), body weight, body mass index (BMI), growth velocity (GV), and the Tanner stage of sexual maturation [6]. In every centre, length/height was measured to the nearest 0.1 cm with a horizontal level- or wall-mounted stadiometer (Harpenden; Crymych, UK); body weight was measured to the nearest 0.1 kg; and BMI was obtained from the weight in kg/length or height in meters squared and expressed as standard deviation score (SDS) with respect to chronological age. Height SDS and BMI SDS were calculated for each value using age- and sex-specific World Health Organization (WHO) growth chart 2007 [7]. Parental height was also collected, when available, to estimate target height (TH), calculated according to the following formulas in cm: [(mother's height + 13) + father's height]/2 in males and [(mother's height − 13) + father's height]/2 in females [8]. Each value of height SDS was also adjusted for TH.

Plasma basal levels of follicle stimulating hormone (FSH), luteinizing hormone (LH) expressed in mUI/ml, oestradiol (pg/ml), or testosterone (ng/dl) were detected through chemiluminescent immunometric assay (Bayer, Germany) at baseline and at each follow-up examination.

In all patients with signs of early pubertal development (groups A and C), GnRH test (LHRH, Ferring, Kiel, Germany; i.v. bolus injection of 100 mcg/m^2^) was performed. An indwelling venous catheter was inserted into an antecubital vein and kept patent by a slow saline infusion. Tests on blood samples were performed before and at 0, 15, 30, 45, 60, and 90 min after LHRH injection to measure LH, FSH, and oestradiol concentrations.

X-ray of the left (or nondominant) wrist and hand was taken for bone age (BA) assessment according to the Greulich & Pyle method at baseline and annually during follow-up in groups A and C. All patients underwent brain MRI.

Data about the therapeutic approach to CPP (Triptorelin) including time interval (28 or 21 days) and the dose of subcutaneous injection (1.875 or 3.75 mg) were gathered from folders of all patients of groups A and C.

The study was approved by the Ethics Committee of the University of Modena and Reggio Emilia (Practice 255/13).

The results were expressed as mean and SDS for quantitative variables or as percentages for qualitative variables. Comparison among groups was performed using the Mann–Whitney nonparametric test (STATISTICA TM software, StatSoft Inc., Tulsa, OK, USA), and comparison among variables of the same group over time was analysed through the Wilcoxon test. The level of significance was set at *p* < 0.05.

## 3. Results

We enrolled 44 Caucasian children with CP: 22 presenting CPP (group A) and 22 without CPP (group B). Group C was composed of 22 Caucasian CPP children without CP.

Table 1 lists baseline features of groups. The majority of CP patients were on AED: 41% and 54% were on levetiracetam, 36% and 54% on valproic acid, 23% and 18% on etosuccimide, 18% and 9% on phenobarbital, 9% and 14% on topiramate, and 4.5% and 14% on gabapentin, respectively, in groups A and B. Most of these patients were on multidrug regimen.

One patient in group A and another one in group B were also on levothyroxine because of hypothyroidism. No other endocrine disease, apart from CPP, was identified in the study population.

No differences in anthropometric parameters were detected among CP patients at the time of diagnosis of CPP (A versus B: length SDS −0.72 ± 1.86 versus −0.53 ± 1.88, *p* = 0.56; BMI SDS 0.67 ± 1.31 versus 0.18 ± 1.58, *p* = 0.35). Performing the analysis according to gender, both females and males with CP and presenting CPP have an increased BMI SDS in comparison to group B; nevertheless, the difference was not statistically significant (A versus B: females 0.58 ± 1.24 versus 0.04 ± 1.71, *p* = 0.40; males 1.11 ± 1.72 versus 0.52 ± 1.33, *p* = 0.71).

**Figure 1:**
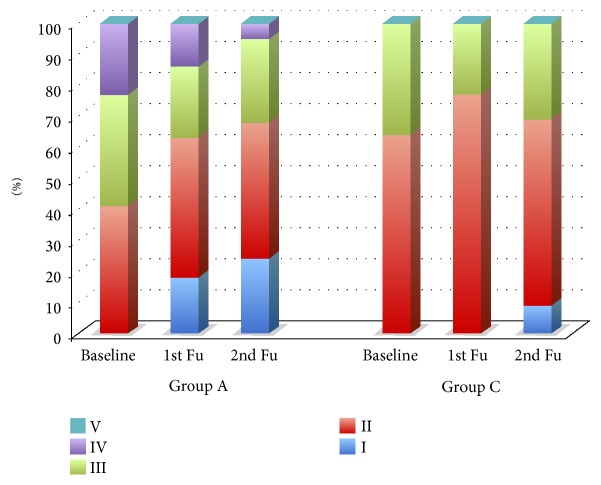
Pubertal stages according to Tanner and Whitehouse [6] in patients with CPP at baseline (diagnosis of CPP) and during GnRHa treatment. Fu: follow-up.

Comparing CPP groups (A versus C), the age of diagnosis of CPP did not differ significantly, even if CP children tended to present CPP earlier (Table 2), and the younger patient with a diagnosis of CPP was a boy with CP aged 2 years and 9 months. At the diagnosis of CPP, CP children were shorter than those in group C, even if length SDS was adjusted for TH (Table 2). Development of sexual maturation was distributed differently in 2 groups according to the Tanner stage (Figure 1). Similarly, basal LH and oestradiol levels were higher in group A than in C, whereas basal FSH and gonadotrophin values after stimulation test did not differ (Table 3).

In group A, two families refused therapy with gonadotrophin-releasing hormone agonist (GnRHa) after the diagnosis of CPP: features of these 2 patients were collected at baseline, but not later. Follow-up lasted 24.80 ± 15.18 (range 7–72) months. At the second follow-up visit, all patients were still on GnRHa. Over time, GnRHa therapy affected differently the growth in the 2 groups: length/height SDS and height SDS adjusted for TH continued to get worse in group A than in group C (Table 2) and at the end of follow-up, the discrepancy among recorded values of height SDS adjusted for TH deteriorated in group A but not in group C (−0.49 ± 0.91 versus 0.21 ± 0.33, *p* = 0.023, resp.) and GV significantly reduced only in group A, even in comparison with group C.

Despite different growth patterns, GnRHa therapy was effective in both groups decreasing basal gonadotropin and oestradiol/testosterone levels (Table 3) and reducing the signs of pubertal progression (Figure 1).

The advancement of bone age in comparison with chronological age was stable over time in group C, while was progressively reduced in group A. In all the study groups, brain MRI did not identify anomalies in hypothalamic-pituitary region.

The dose of GnRHa started at 1.875 mg only in 2 patients belonging to group A according to their age and/or weight. The remaining patients in group A and all patients of group C were on Triptorelin at the dose of 3.75 mg. The time interval between injections corresponded to 21 days for 32% of group A patients, but for none of group C (on regular monthly injection).

## 4. Discussion

CP is a complex condition that affects individuals differently. Its treatment and management are challenging because families, caregivers, and clinicians have to face, besides motor, cognitive, and neurologic disorders, also nutritional and respiratory concerns, infections, osteoporosis, and more rare endocrine problems [9]. Data from Worley et al. described the physiological pattern of pubertal development observed in 207 CP children with moderate to severe motor impairment: girls seemed to enter puberty earlier but tended to mature over a longer period of time, menstruating later; boys followed more regular patterns but tended to start earlier [4]. The evaluation of CPP in CP and its prevalence lay outside the scope of the mentioned study. A higher prevalence of variable degrees of early sexual maturation was described among 161 girls with neonatal encephalopathy in comparison to general population (4.3% versus 0.6%) [3]. However, limited data have been published on this topic [1] and most of the already published papers evaluated wide populations of children with variable degree of neurodevelopmental disabilities, and not exclusively CP [1, 3]. Our study focused on the association between CP and CPP, even if, because of its design, no data about the prevalence of their association could be deduced.

Precocious puberty is reported 8 times more frequently in girls than in boys, and underlying organic disorders are more commonly discovered in males [10]. Strikingly, in our study, 4 of 22 children with CP and CPP were male, whereas only females composed group C.

Even if ages at baseline did not differ between groups A and C (Table 2), our results evoke a precocity of the age in the onset of CPP in CP than in the general population. Moreover, CPP seems to progress rapidly in CP: at the diagnosis of CPP, a great percentage of CP patients presented a more advanced pubertal stage (Figure 1) and higher levels of basal LH and oestradiol were documented in group A than in group C (Table 3). These data let us suppose a more intense activation of the hypothalamic-pituitary-gonadal axis in CP that might be due to a loss of normal childhood hypothalamic inhibition of pituitary gonadotropins. Severe brain damage and AED may affect a number of neurotransmitter pathways involved in gonadotropin control [11]. In our study, brain neuroimaging was performed in all CP patients, without finding abnormalities in hypothalamic-pituitary region. Moreover, the majority of CP patients were on AED treatment independently from CPP.

In our cohort of patients, the lack of statistical difference between the age of diagnosis of CPP in group A and in C could be explained by the hypothesis that in CP, even in the presence of signs of pubertal developmental, the diagnosis of CPP could be delayed: first of all, parents or caregivers could underestimate the problem due to the complexity and severity of the general physical condition of the patient and, additionally, a growth failure could partially mislead the initial picture. In fact, as already reported in literature, children with CP are usually affected by growth impairment [12, 13]. In CP, growth retardation might have a multifactorial aetiology, and it could be associated with nonnutritional factors: the type of movement disorder, the severity of CP, mainly regarding self-feeding and walking capacity, the level of physical activity limitation, and endocrine disorders seem to act synergistically. Typical pubertal growth spurt appears to be significantly diminished in CP children [14]. Other descriptive data confirm a steady height velocity before and during puberty in CP [15]. Lower concentrations of insulin-like growth factor 1 (IGF-1) and of growth hormone (GH) were described in girls with CP, with a similar trend for boys, supporting the hypothesis of a dysfunction of the hypothalamic-pituitary axis [16]. In our study, the observation of a significant reduction of growth velocity over follow-up and of a progressive increase of discrepancy between patient's height SDS and familiar target height over time (Table 2) supports the hypothesis of a complex growth disorder in CP.

The relationship between sexual maturation and nutritional status has to be considered in CP. More advanced sexual maturation was associated with more body fat in girls, but with less body fat in boys [4]. Our data did not support the aetiological role of BMI in developing CPP in CP: no changes in BMI SDS were documented between groups A and C over follow-up and, more interestingly, between groups A and B at the time of diagnosis of CPP. Nevertheless, we cannot consider BMI as a reliable indicator of nutritional status. The use of BMI in a traditional manner in children with reduced muscle mass, as happened in CP, could inadvertently result in an overestimation of their nutritional status [17]. Other tools such as waist and hip circumferences, skin-fold thickness together with serum leptin levels should be measured in CP children in future studies [18].

Patients with CP and only minimal motor dysfunction can be expected to achieve weights and heights close to those of sex- and age-matched general population, while the discrepancy increases in patients with significant motor dysfunction. Therefore, the use specific growth charts are encouraged [15, 19]. In our study, we decide to collect CP statural data instead of segmental measurements and to compare them to standard chart in order to better underline differences among groups. Moreover, we agree with the US Centre for Disease Control and Prevention: these population-specific growth charts describe “how CP patients grow” rather than “how they should grow” and, therefore, could minimize their growth disturbance [20]. CP is different from some genetic conditions that are known to alter growth. CP is a condition that only has “the potential to alter growth” [14].

For families (taking care of children who present severe motor impairment), the reduction in final growth, occurring usually in CPP when untreated, could appear even convenient: lifting, transferring, and transporting child may be easier at a smaller size. Considering the international debate about the theme of attenuating growth in children with serious disabilities [21–23], we intentionally avoid dealing with any ethical issues in this paper: any therapeutic decision should consider the peculiarity of each case and could be different for every family. Generally, in childhood, the major goals in the medical management of CPP include halting the advancement of bone age, arresting secondary sexual development, and optimizing adult height. Other treatment issues could include the child's emotional changes to CPP and the family's worries [24]. In our study, we document that during follow-up, despite GnRHa therapy, growth failure in CP seemed to worsen. This finding imposes clinicians to redefine the realistic objectives of GnRHa when treating CP patients and/or to identify other therapeutic options to optimize results. In this prospective, other efforts should be done to understand the aetiology of CPP in CP. In fact, considering the multiple neurotrophic effects of GH-IGF-1 axis in both central and peripheral nervous system, some cognitive disorders characterizing CP have been documented to reverse by GH replacement [25]. Similarly, other hormones, especially reproductive steroids and oestradiol, have some neuroactive properties and, at the same time, epileptic substrate seems to be susceptible to neuroactive sexual steroid effects [26]. As happened for hypothalamic gelastic seizure [27] and for catamenial epilepsy [28], we can now only speculate that GnRHa could not only influence the development of pubertal progression but also help in controlling the potential concomitant deterioration of neurologic symptoms as seizures in CP. Because our study did not include data on these neurological aspects (e.g., frequency and type of seizure, plasmatic levels of AEDs), further specific studies are needed.

## 5. Conclusion

Our data clearly demonstrated the challenge of the diagnosis and the specific peculiarities of CPP in CP. Firstly, health-care providers have to be vigilant in screening for early pubertal changes in children with CP despite the presence of growth failure; then, they have to critically select the correct tools to detect anthropometric and nutritional status and, finally, they have to understand and redefine the purposes of the management of CPP in CP to better support parents in order to ensure the best therapeutic choice for each patient.

## Figures and Tables

**Table 1:** Baseline features of groups A, B, and C.

	Group A	Group B	Group C
Num. (F/M)	22 (18/4)	22 (17/5)	22 (22/0)
Baseline age (years) (mean ± SDS; range)	6.42 ± 1.74 (2.74–8.90)	6.63 ± 1.85 (2.10–9.70)	7.02 ± 0.79 (5.20–7.96)
Gestational age (weeks) (mean ± SDS)	36.93 ± 4.61	37.94 ± 4.11	39.33 ± 0.70
Apgar 1 min (mean ± SDS)°	4.58 ± 3.02	3.23 ± 2.24	9.01 ± 0.54
Apgar 5 min (mean ± SDS)°	6.25 ± 2.56	5.00 ± 1.80	9.50 ± 0.22
Birth weight (kg) (mean ± SDS)	2.65 ± 0.85	3.04 ± 0.84	3.33 ± 0.35°
GMFCS (III, IV, V) (%)	68, 27, 5	73, 18, 9	NA
Use of antiepileptic medication (%)	77%	100%	0%

°Significant difference between groups A and C.

**Table 2:** Longitudinal evaluation of anthropometric features of patients with CPP at baseline (diagnosis of CPP) and during GnRHa treatment. H-TH SDS: height SDS adjusted for target height (TH).

	Baseline		1st follow-up visit°		2nd follow-up visit°	
	Group A	Group C	Group A	Group C	Group A	Group C
Chronological age (CA)	6.42 ± 1.74	7.02 ± 0.79	7.50 ± 1.75°	8.02 ± 0.90°	8.59 ± 2.15°	8.69 ± 0.55°
Height SDS	−0.72 ± 1.86	0.75 ± 1.46^^^	−0.96 ± 1.86	0.87 ± 1.38^^^	−1.32 ± 1.63	0.76 ± 1.70^^^
H-TH SDS	−0.63 ± 1.94	1.56 ± 1.38^^^	−0.72 ± 1.90	1.68 ± 1.26^^^	−1.07 ± 1.83	1.48 ± 1.42^^^
Discrepancy between bone age (BA) and CA	1.74 ± 1.18	1.74 ± 1.57	1.07 ± 1.01°	1.65 ± 0.98	0.72 ± 0.65°	0.89 ± 0.43
Growth velocity (cm/years)	6.67 ± 1.22	9.1 ± 0.99	5.61 ± 1.99	6.70 ± 1.34	4.51 ± 3.04°	6.60 ± 1.30^^^
BMI SDS	0.67 ± 1.31	0.45 ± 0.82	0.15 ± 1.54°	0.57 ± 1.02	0.52 ± 1.21	0.24 ± 0.98

°Significant difference in the same group from baseline. ^^^Significant difference between the groups at the specific time point.

**Table 3:** Longitudinal evaluation of biochemical data of patients with CPP at baseline (diagnosis of CPP) and during GnRHa treatment.

	Baseline		1st follow-up visit		2nd follow-up visit	
	Group A	Group C	Group A	Group C	Group A	Group C
LH (mUI/ml)	2.54 ± 2.23	0.49 ± 0.50^^^	0.58 ± 0.56	0.28 ± 0.17	0.48 ± 0.55	0.28 ± 0.15
FSH (mUI/ml)	5.34 ± 5.96	3.44 ± 1.41	1.86 ± 1.78	0.78 ± 0.57	2.65 ± 2.18	0.47 ± 0.25
Oestradiol (pg/ml)	27.13 ± 16.92	12.65 ± 6.94^^^	12.36 ± 6.72	11.93 ± 2.94	11.13 ± 3.02	11.28 ± 2.21

^^^Significant difference between the groups at the specific time point.
